# Appetite Stimulant and Anti-Emetic Effect of Mirtazapine Transdermal Ointment in Cats Affected by Lymphoma Following Chemotherapy Administration: A Multi-Centre Retrospective Study

**DOI:** 10.3390/ani12020155

**Published:** 2022-01-09

**Authors:** Livia Ferro, Stefano Ciccarelli, Giacomo Stanzani, Lisa Nappi, Francesca Angelini, Chiara Leo

**Affiliations:** 1 Anicura Istituto Veterinario Novara, 28060 Granozzo con Monticello, Italy; chiara.leo@anicura.it; 2 Department of Veterinary Medicine, University of Bari “Aldo Moro”, 370100 Valenzano, Italy; stefano.ciccarelli@uniba.it; 3 Dick White Referrals, Cambridgeshire CB8 0UH, UK; giacomo.stanzani.16@ucl.ac.uk; 4 Anicura Centro Oncologico Veterinario, 40037 Sasso Marconi, Italy; lisa.nappi@anicura.it; 5 Anicura Clinica Veterinaria CMV, 21100 Varese, Italy; francesca.angelini@anicura.it

**Keywords:** mirtazapine, feline, chemotherapy, cancer, transdermal, toxicity, anti-emetic

## Abstract

**Simple Summary:**

Feline cancer patients’ owners are increasingly willing to undertake oncologic treatment, such as chemotherapy. Concerns regarding worsening quality of life are common since chemotherapy could cause toxicities, such as vomiting, nausea, anorexia, and consequently weight loss. In humans, mirtazapine effectively prevents chemotherapy-induced nausea and vomiting, improving the quality of life in people receiving chemotherapy. Recently, the use of mirtazapine transdermal ointment has been evaluated in cats with non-cancer diseases. This study describes the use of transdermal mirtazapine administration in cats diagnosed with lymphoma and receiving chemotherapy. Patients included in the study did not receive any prophylactic anti-emetics other than transdermal mirtazapine. Data regarding patients, type of chemotherapy, and incidence of weight loss and gastrointestinal toxicities were retrospectively evaluated. Transdermal mirtazapine was well tolerated, and substantial weight loss was not observed in the 14 days following chemotherapy administration. These results support further studies assessing the impact of mirtazapine in preventing chemotherapy-induced gastrointestinal toxicity in cats.

**Abstract:**

In humans, mirtazapine can prevent chemotherapy-induced nausea and vomiting (CINV) and improve cancer patients’ quality of life (QoL). This drug is being increasingly used as an appetite stimulant in cats. The hypothesis of this retrospective study was that mirtazapine could reduce the incidence of CINV and weight loss in feline patients affected by lymphoma. The objectives were to report the use of mirtazapine transdermal ointment and assess the incidence of gastrointestinal (GI) toxicity and weight loss in cats diagnosed with lymphoma and receiving chemotherapy. Transdermal mirtazapine was topically administered to the inner surface of the pinna (2 mg/cat/daily) for 14 days following chemotherapy administration. Data recorded from 20 patients were collected. Different grades of GI toxicity were shown in 8/20 (40%) patients. Body weight (BW), body condition score (BCS), and muscle condition score (MCS) improved in 12/20 (60%), 6/20 (30%), and 2/20 (10%) cats, respectively. Mirtazapine-induced adverse events (AEs) occurred in 4/20 (20%) cats and did not require mirtazapine discontinuation. Substantial weight loss was not encountered, suggesting that patients had an adequate food intake after chemotherapy administration. Transdermal mirtazapine ointment was considered safe and well tolerated.

## 1. Introduction

Veterinary oncologists constantly need to balance the toxicity of anti-cancer treatments against their effectiveness, as maintaining a good patients’ quality of life (QoL) is a milestone for professional ethics and the ultimate goal for owners. Nausea, vomiting and decreased appetite due to antineoplastic treatments can affect this QoL and influence the owners’ decisions to engage in appropriate cancer treatments or decline them [1].

Chemotherapy-induced nausea and vomiting (CINV) have a multifactorial aetiology involving different neuromediators and receptors located in the central nervous system and gastrointestinal (GI) tract [2]. Substance P and neurokinin-1 (NK-1) receptors, dopamine and its receptors, and serotonin (5-hydroxytryptamine or 5-HT3) and its receptors are all involved in CINV pathophysiology in both humans and pets [3]. In particular, the peripheral 5-HT3-related pathway is responsible for acute emesis because its activation occurs in the first 24 h after chemotherapy administration, whereas the central NK-1-related pathway is implicated in delayed CINV [3]. Receptors involved in CINV represent the target for drugs such as maropitant, ondansetron, and metoclopramide, administered to prevent GI adverse events (AEs) in patients receiving chemotherapy.

Recently, mirtazapine, a tetracyclic antidepressant, has gained popularity as an appetite stimulant and anti-emetic in cats with decreased appetite and weight loss associated with chronic kidney disease (CKD) and other disorders [4,5,6]. Mirtazapine has been shown to block pre-synaptic α2-receptors by increasing serum release of serotonin and norepinephrine. Moreover, mirtazapine is a potent antagonist of H1 histamine receptors, 5-HT2A, 5-HT2C, and 5-HT3 serotonin receptors and therefore plays a role in nausea and vomiting control and prevention [7,8]. The mechanism of action for appetite stimulation is unclear. It is probably due to its antagonistic action on the 5-HT2C receptors, which are responsible for appetite inhibition, and on the H1 receptors involved in appetite regulation [9,10].

In human cancer patients, it has been demonstrated that mirtazapine can achieve a significant clinical benefit in treating CINV, thereby improving QoL in people receiving chemotherapy [7,11,12]. A Phase II clinical trial in humans experiencing anorexia and cachexia associated with cancer demonstrated increased body weight (BW) and appetite after mirtazapine administration [13].

Mirtazapine has been historically available as oral tablets, and only in 2018 a transdermal ointment has been approved by the Food and Drug Administration (FDA). The ointment is currently approved for weight loss control in cats at the dose of 2 mg/cat once daily for 14 days. Efficacy and safety have been evaluated in a multi-centre, double-blind, placebo-controlled, randomised clinical study that included cats with unintended weight loss but excluded feline patients affected by neoplastic disorders, severe kidney disease (International Renal Interest Society Stage 4 or serum creatinine > 5.0 mg/dL) or with a BW < 2 kg [5]. It has been reported that cats receiving mirtazapine transdermal ointment showed significant BW gain (+3.9%) compared to patients included in the placebo group (+0.4%) (*p* < 0.0001) [5]. Treatment was well-tolerated, and the most common adverse effect (AE) was mild erythema at the application site, seen in 10.4% of mirtazapine-treated cats [5].

A study evaluating mirtazapine toxicity in 84 cats reported that AEs were more likely associated with high doses of mirtazapine. The most common AEs were vocalization (56%), agitation (31%), and vomiting (26.2%), encountered more frequently with over-dosage (average dose > 2.56 mg/kg) [14].

A PubMed search of the keywords “mirtazapine, cat and/or feline, cancer, neoplasia, lymphoma, chemotherapy” and their combinations was conducted. No studies assessing mirtazapine’s appetite stimulating and anti-emetic properties in cancer-bearing cats receiving chemotherapy were found. Since appetite and vomiting are parameters perceived by owners as important in influencing QoL [15], mirtazapine could be helpful for GI toxicity prevention and, subsequently, QoL improvement.

The hypothesis of this study was that mirtazapine, as an appetite stimulant and 5-HT3 antagonist, would be well tolerated and could prevent CINV and weight loss, improving QoL in cats affected by lymphoma. This retrospective study aimed to describe the use of mirtazapine transdermal ointment in feline patients with lymphoma receiving chemotherapy and evaluate the incidence of chemotherapy-associated GI toxicity in this population.

## 2. Materials and Methods

This multi-centre retrospective study included client-owned cats diagnosed with lymphoma and receiving chemotherapy. Clinical data were collected from 4 different veterinary hospitals in Italy (Anicura Istituto Veterinario of Novara, Anicura Centro Oncologico Veterinario of Sasso Marconi, Anicura Clinica Veterinaria CMV of Varese, and Department of Veterinary Medicine of University of Bari) between May and October 2021.

Cats met inclusion criteria if a cytological or histological diagnosis of lymphoma was made and if they received chemotherapeutic agents and trans-dermal mirtazapine. The administration of systemic corticosteroids, required by the chemotherapy protocol, and prophylactic antibiotics was not considered an exclusion criterion. Cats were excluded if they showed a BW < 2.0 kg or showed loss of appetite, anorexia or nausea at presentation prior to receiving chemotherapy.

If patients presented persistent anorexia, nausea, or vomiting despite mirtazapine administration at any point during the chemotherapy protocol and they receive maropitant, if considered indicated by the primary clinician, they were not excluded from the study.

The administration of any additional medications to prevent nausea and vomiting or stimulate the appetite before or after the administration of chemotherapy resulted in exclusion from the study. These drugs included ondansetron, cyproheptadine, metoclopramide, diazepam, phenothiazines, marinol and oxazepam.

Mirtazapine transdermal ointment (Mirataz^®^ [Dechra Veterinary Products, Shrewsbury, UK]) was topically applied to the inner surface of the pinna at 2 mg/cat once daily for 14 days. Treatment started the same day of chemotherapy administration. Other mirtazapine formulations, routes of administration or doses resulted in patient exclusion.

Data regarding age, sex, breed, tumour type, chemotherapy protocol, and additional medications were collected. The BW of each cat was measured on the first day of mirtazapine administration (day 1) and the day after the last mirtazapine administration (day 15) from 4 weight scales, one for each clinic. Information regarding physical examination, World Small Animal Veterinary Association (WSAVA) body condition score (BCS), and WSAVA muscle condition score (MCS) were recorded on day 1 and day 15 [16]. Cats were fasted prior to each clinical examination.

Chemotherapy-associated GI toxicity was assessed based on VCOG-CTCAE (Veterinary co-operative oncology group—common terminology criteria for adverse events) grading system for nausea, vomiting and anorexia (Table 1) [17].

Mirtazapine-associated AEs were evaluated by referring to those reported in veterinary scientific literature and known to be related to mirtazapine administration [14].

All patients’ owners received an owner log to record chemotherapy-associated GI toxicity and mirtazapine’s AEs. A signed consent form was requested.

Descriptive statistics were performed on the following factors: age, gender, breed, BW, type of chemotherapy, additional treatments, GI toxicity grade for nausea, vomiting and anorexia, and AEs.

Continuous variables were tested for normality and reported as mean (± SD) if normally distributed, or median (range minimum-maximum) if not normally distributed. Categorical variables were reported as percentages. Body weight changes were calculated between day 1 and day 15 timepoints. An increase or decrease in BW ≥ 0.1 kg identified a BW gain or BW loss, respectively.

## 3. Results

Seventy-six cats diagnosed with neoplasia and requiring chemotherapy were seen during the study period. Of these patients, 13/76 (17%) patients’ owners declined chemotherapy, 24/76 (32%) received chemotherapy without transdermal mirtazapine treatment, 5/76 (7%) received chemotherapy and transdermal mirtazapine but had different neoplasms, 4/76 (5%) had BW < 2.0 kg, and 10/76 (13%) were anorexic at presentation.

The other 20/76 (26%) cats met the inclusion criteria. None of these patients was excluded during the 15 days of mirtazapine administration. Information regarding age, sex, breed, anatomic location of lymphoma, type of chemotherapy, additional treatments, BW, BCS, and MCS recorded at day 1 and day 15 is shown in Table 2.

Eight out of the 20 (40%) cats included in the study showed chemotherapy-associated GI toxicity. Among these, 3/8 (38%) required maropitant administration. The 3 cats requiring maropitant had received doxorubicin, lomustine, and vincristine. Nausea, vomiting, and anorexia were reported in 5/8 (63%), 2/8 (25%), and 7/8 (88%) cats, respectively. Among cats with GI toxicities, 4/8 (50%) and 1/8 (13%) showed respectively grade 1 and grade 2 nausea; 2/8 cats (25%) showed grade 1 vomiting; grade 1 and grade 2 anorexia were reported in 4/8 (50%) and 3/8 (38%) cats, respectively (Table 3).

Three of 6 (50%) cats receiving lomustine, 1/1 patient receiving doxorubicin, and 4/7 (57%) cases receiving vincristine showed GI toxicity.

Among cats with grade 1 nausea, 2/4 (50%) received lomustine, 1/4 (25%) doxorubicin, and 1/4 (25%) vincristine. The cat manifesting grade 2 nausea had received lomustine. The 2 cats with grade 1 vomiting had received doxorubicin and lomustine. Among patients with grade 1 anorexia, 1/4 (25%) received lomustine, and 3/4 (75%) received vincristine. In 2/3 (67%) and 1/3 (33%) of cats with grade 2 anorexia lomustine and doxorubicin were respectively administered. Among the 8 cats with lymphoma experiencing GI toxicity, 4/8 (50%) had a GI lymphoma, 3/8 (38%) had mediastinal lymphoma, and 1/8 (12%) had pharyngeal lymphoma.

Twelve out of 20 cats (60%) showed a BW gain (≥0.1 kg) between day 1 and day 15. One out of 20 (5%) had a decrease in BW, while 7/20 (35%) cats did not show differences in BW.

The overall median percent change in BW from day 1 to day 15 was +2% (range −11%–+21%).

Median BCS was 4 (range 2–6) at day 1 and 5 (range 3–6) at day 15, and improvement in BCS was seen in 6/20 (30%) cats. Muscle condition score was considered increased in 2/20 (10%) cats.

Mirtazapine-associated AEs were seen in 4/20 (20%) cats. Among these, 2/4 (50%) cases had pruritus of the inner surface of the pinna, and 2 (50%) patients showed vocalisation. These AEs were considered minor, so the primary clinicians did not deem it necessary to discontinue treatment. All the included patients had received the standard dose of transdermal mirtazapine (Table 4).

## 4. Discussion

To the best of the authors’ knowledge and following the authors’ literature review, this is the first study to describe the use of mirtazapine as appetite-stimulating and anti-emetic in cats affected by lymphoma and receiving chemotherapy.

Mirtazapine appeared safe and well tolerated in our population. The lack of substantial weight loss suggests that adequate food intake was maintained throughout, and the effects of GI toxicity on appetite were limited.

In humans, clinical trials evaluated the use of mirtazapine for cancer-related anorexia and cachexia (CRCA) and as a secondary prophylactic for nausea related to highly emetogenic chemotherapy administration [12,13]. These studies highlighted that mirtazapine is a promising drug for the treatment of CRCA, resulting in good tolerance and significant benefit in QoL’s human patients receiving chemotherapy.

In veterinary medicine, mirtazapine’s properties in stimulating appetite, promoting weight gain, and reducing vomiting have been reported in cats with CKD [4,5].

To date, studies regarding the incidence of GI toxicity in cats receiving chemotherapy with or without anti-emetic drugs are still lacking. In our study population, 60% of cases did not show GI toxicity. In those patients experiencing nausea, vomiting and anorexia, clinical signs were mild and self-limiting in most cases. Only 3/8 (37%) cats experienced persistent GI toxicity despite transdermal mirtazapine application and required maropitant administration.

It is important to note that vomiting is one of the most common AEs (26% of cases receiving a dose between 0.73 and 5.38 mg/kg) following oral mirtazapine administration [14], but has not been reported with topical mirtazapine formulations. For this reason, we consider it unlikely that the vomiting seen in our study population occurred as a consequence of transdermal mirtazapine administration.

In our study population, signs of nausea, anorexia, and vomiting were encountered in 4/7 (57%) cats receiving vincristine. Tzannes et al. evaluated GI toxicity in cats with lymphoma receiving a COP protocol (vincristine, cyclophosphamide and prednisolone) and reported loss of appetite, vomiting, and weight loss in 29%, 23%, and 13% of cases, respectively [18]. In our study, among all cats receiving vincristine, 1/7 (14%) cats showed grade 1 nausea, and 3/7 (42%) cases had grade 1 anorexia. Weight loss was not found in cats receiving vincristine. These data cannot be accurately compared since cats in the previous study also received cyclophosphamide in association with vincristine. However, previous reports that included cats receiving low-dose cyclophosphamide showed grade 1 GI toxicity in 16–17% of cases [19,20]. Only 1 cat received cyclophosphamide in our study and it did not have GI toxicity, but conclusions cannot be drawn due to the small sample size.

All patients receiving doxorubicin showed mild GI toxicity (grade 1 anorexia, vomiting, and nausea) in our study. In contrast, a previous study reported that 40 tumour-bearing cats receiving 129 doses of doxorubicin at the dose of 1 mg/kg reported 8.5% of cats showing grade 1 vomiting, 9.3% grade 2, and 0.8% grade 3, without significant differences compared to another group of 20 tumour-bearing cats who received doxorubicin at the dose of 25 mg/m^2^ [21]. Moreover, another study reported a loss of appetite as the most common toxicity in cats affected by lymphoma receiving doxorubicin, observed in 47% of all cases and being severe in 26% of cases [22]. In our study, 1/3 (33%) patients receiving doxorubicin needed adjuvant maropitant administration due to persistent GI toxicity; however, this was not considered severe.

To date, several studies evaluate the tolerability of lomustine in cats with various neoplasias, reporting that it is usually well-tolerated, with a low incidence of GI toxicity [23,24,25,26]. This is in contrast with data observed in our study, where 3/6 (50%) cats receiving lomustine showed GI toxicities (grade 1 nausea and anorexia in a cat, grade 2 nausea and anorexia a cat, and grade 1 nausea and vomiting and grade 2 anorexia in another patient), and one required maropitant administration. However, previous studies do not report information regarding the use of anti-emetics or appetite stimulants.

Cats in our study showed a BW improvement of +2% (range −11%–+21%) at day 15, compared to baseline. These results are similar to what was reported in a recent pivotal mirtazapine field safety and effectiveness study. This study included 177 cats with unintended weight loss and reported a +3.9% BW change in cats receiving mirtazapine transdermal ointment [5]. This increase was statistically significant compared to the placebo group, where BW change was only +0.4% [5].

It should be noted that in addition to mirtazapine, our study population also received chemotherapy and corticosteroids, which could have helped clinical improvement and, consequently, increase in appetite and BW.

Body condition score and MCS improved in 6/20 (30%) and 2/20 (10%) cats, respectively. These findings are similar to those reported in a recent study, including 19 cats with CKD that received compounded transdermal mirtazapine at two different doses (1.88 and 3.75 mg) [6].

Both BCS and MCS are closely related to patients’ BW, which is a critical feature taken into consideration by owners in their pets’ QoL evaluation. Indeed, one of the most important factors influencing owners’ decision to use chemotherapy in terminally ill pets is weight loss, which is considered unacceptable [1]. For this reason, transdermal mirtazapine could be a valuable solution for veterinary practitioners, to improve both pets’ QoL and owners’ compliance by managing GI toxicity and BW in cats with lymphoma receiving chemotherapy. This could be facilitated by the handling of the trans-dermal compound, which owners usually prefer since it is easier to administer compared to oral medications.

Moreover, it is important to consider how BCS and MCS changes could reflect metabolic alterations associated with paraneoplastic cachexia, which negatively affects prognosis, both in humans and in feline cancer patients [27,28]. It has been demonstrated that tumour-bearing cats with a BCS < 5 had a significantly shorter survival time (3.3 months) compared to those with a BCS > 5 (16.7 months) [28]. Therefore, it is possible to hypothesise that through medical strategies, such as mirtazapine administration, improvement in BCS, MCS, and consequently in prognosis, could be achieved.

It should be noted that the evaluation of MCS is subjective and therefore presents some limitations, in particular in patients with paraneoplastic cachexia and sarcopenia, and over the short period of time of our study (15 days).

Mirtazapine-associated AEs were encountered in 4 of 20 of our patients. Among them, 2 cats showed pruritus of the inner surface of the pinna, and 2 cases showed vocalisation. These symptoms were mild and self-limiting. Haematology and biochemistry were not available for all patients; for this reason, haematological toxicities could not be evaluated.

According to the veterinary literature, AEs induced by the administration of mirtazapine at standard doses are usually acceptable and self-limiting. A previous study reported application site erythema as the most common AE in cats receiving trans-dermal mirtazapine at 2 mg/cat [5]. Another study, assessing toxicity of mirtazapine in 84 cats, reported that the most common AEs were vocalization (56.0%), agitation (31.0%), vomiting (26.2%), abnormal gait/ataxia (16.7%), restlessness (14.3%), tremors/trembling (14.3%), hypersalivation (13.0%), tachypnea (11.9%), tachycardia (10.7%), and lethargy (10.7%). In most cases (70.2%) ingestion was considered accidental and the mean dose of mirtazapine in these patients was >2.4 mg/kg, which is higher than a standard dose [14].

According to our study and the previous literature, the application of 2 mg/cat of mirtazapine transdermal ointment appears to be generally safe and well-tolerated [5,6,29].

This study has several limitations, including its retrospective nature, the small number of patients, and the absence of a control group. Moreover, patients included in this study received different chemotherapeutic agents which could have resulted in a variable incidence of GI toxicities. A more homogeneous treatment protocol, in terms of type of chemotherapy and concurrent therapies, would have been preferrable. Future standardised prospective clinical studies may help overcome these limitations.

## 5. Conclusions

This is the first study describing the use of transdermal mirtazapine in cats affected by lymphoma receiving chemotherapy.

In this retrospective study, the use of transdermal mirtazapine appeared safe and well tolerated. Patients did not show substantial weight loss, suggesting an adequate food intake during the two weeks following chemotherapy administration.

Adverse effects were mild, self-limiting, and acceptable. These results agree with previous studies conducted in patients with non-cancer diseases. Our study support further randomised, placebo-controlled clinical trials, to assess usefulness of transdermal mirtazapine in preventing chemotherapy-associated GI toxicities, thus improving QoL of feline oncologic patients, and consequently owners’ compliance and satisfaction.

## Figures and Tables

**Table 1:** Veterinary Cooperative Oncology Group-Common Terminology Criteria for Adverse Events (VCOG-CTCAE v2) following investigational therapy in dogs and cats. LeBlanc AK, et al.; Vet Comp Oncol. 2021) [17].

**Anorexia**	
Grade 1	Coaxing or dietary change required to maintain appetite
Grade 2	Oral intake altered (≤3 days) without significant weight loss; oral nutritional supplements/appetite stimulants may be indicated
Grade 3	Of >3 days duration; associated with significant weight loss (≥10%) or malnutrition; IV fluids, tube feeding or force feeding indicated
Grade 4	Life-threatening consequences; TPN indicated; >5 days duration
Grade 5	Death
**Nausea**	
Grade 1	Loss of appetite without alteration in eating habits
Grade 2	Salivation or ‘smacking of lips’ <3 days, grade 2 anorexia
Grade 3	Salivation or ‘smacking of lips’ >3–5 days, grade 3 anorexia
Grade 4	Salivation or ‘smacking of lips’ >5 days, grade 4 anorexia
**Vomiting**	
Grade 1	<3 episode in 24 h, medical intervention not indicated
Grade 2	3–10 episodes in 24 h; <5 episodes/day for ≤48 h; parenteral fluids (IV or SC) indicated ≤48 h; medications indicated
Grade 3	Multiple episodes >48 h and IV fluids or PPN/TPN indicated >48 h
Grade 4	Life-threatening (e.g., haemodynamic collapse)
Grade 5	Death

**Table 2:** Patients information.

**Age**	**Years**	**Chemotherapy**	**n (%)**
Mean ± SD	10.3 (±3.2)	Cyclophosphamide	1 (5.0)
Median (range)	11 (2–16)	Chlorambucil	5 (25.0)
**Sex**	**n (%)**	Doxorubicin	1 (5.0)
Male Neutered	13 (65.0)	Lomustine	6 (30.0)
Female Spayed	7 (35.0)	Vincristine	7 (35.0)
**Breed**	**n (%)**	**Additional treatment**	**n (%)**
Chartreux	2 (10.0)	Antibiotics	5 (25.0)
Domestic shorthair cat	16 (80.0)	Prednisolone	18 (90.0)
Ragdoll	1 (5.0)		
Birman	1 (5.0)		
**Lymphoma anatomic location**			**n (%)**
Gastrointestinal			12 (60.0)
Mediastinal			3 (15.0)
Multicentric			2 (10.0)
Splenic			1 (5.0)
Pharyngeal			1 (5.0)
Nasal			1 (5.0)
**Body weight—day 1**	**Kg**	**Body weight—day 15**	**Kg**
Mean ± SD	4.5 (±1.1)	Mean ± SD	4.6 (±1.1)
Median (range)	4.6 (2.1–6.4)	Median (range)	4.7 (2.2–6.4)
**BCS—day 1**	**n (%)**	**BCS—day 15**	
2	1 (5.0)	2	-
3	2 (10.0)	3	4 (20.0)
4	9 (45.0)	4	5 (25.0)
5	7 (35.0)	5	10 (50.0)
6	1 (5.0)	6	1 (5.0)
**MCS—day 1**	**n (%)**	**MCS—day 15**	**n (%)**
Marked muscle wasting	1 (5.0)	Marked muscle wasting	-
Moderate muscle wasting	1 (5.0)	Moderate muscle wasting	1 (5.0)
Mild muscle wasting	1 (5.0)	Mild muscle wasting	3 (15.0)
Normal muscle mass	17 (85.0)	Normal muscle mass	16 (80.0)

**Table 3:** Incidence of AEs.

**Type of AE**	**n (%)**
No AEs	16 (80.0)
Pruritus	2 (10.0)
Vocalization	2 (10.0)

**Table 4:** Incidence of GI toxicities.

**Nausea**	**n (%)**
No	15 (75.0)
Grade 1	4 (20.0)
Grade 2	1 (5.0)
**Vomiting**	**n (%)**
No	18 (90.0)
Grade 1	2 (10.0)
**Anorexia**	**n (%)**
No	13 (65.0)
Grade 1	4 (20.0)
Grade 2	3 (15.0)

## Data Availability

The data presented in this study are available on request.
